# Supplementary material for: Hot spot prediction in protein-protein interactions by an ensemble system
Source: BMC Syst Biol. 2018 Dec 31;12(Suppl 9):132. doi: 10.1186/s12918-018-0665-8 (PMC6311905; doi:10.1186/s12918-018-0665-8)
Supplement: Supplementary file 2 — All relevant MATLAB code. A simple Matlab implement of our predictor. (ZIP 19,742 kb) [file 12918_2018_665_MOESM2_ESM.zip › Additional file 2/Read ASA/ASA website/SKEMPI/2VLJE.htm]

 NetSurfP - Protein Surface Accessibility and Secondary Structure Predictions

  

|  |  |  |
| --- | --- | --- |
|  |  | NetSurfP - Protein Surface Accessibility and Secondary Structure Predictions  Technical University of Denmark |

  


---

```
# For publication of results, please cite:
# A generic method for assignment of reliability scores applied to solvent accessibility predictions.
# Bent Petersen, Thomas Nordahl Petersen, Pernille Andersen, Morten Nielsen and Claus Lundegaard
# BMC Structural Biology 2009, 9:51 doi:10.1186/1472-6807-9-51
#
# Column 1: Class assignment - B for buried or E for Exposed - Threshold: 25% exposure, but not based on RSA
# Column 2: Amino acid
# Column 3: Sequence name
# Column 4: Amino acid number
# Column 5: Relative Surface Accessibility - RSA
# Column 6: Absolute Surface Accessibility
# Column 7: Z-fit score for RSA prediction
# Column 8: Probability for Alpha-Helix
# Column 9: Probability for Beta-strand
# Column 10: Probability for Coil
E G  2VLJE                  1    0.803  63.235  -0.603   0.003   0.003   0.994
B I  2VLJE                  2    0.271  50.061  -0.859   0.004   0.138   0.858
E T  2VLJE                  3    0.564  78.171  -0.299   0.005   0.262   0.733
B Q  2VLJE                  4    0.242  43.275   0.060   0.020   0.205   0.775
E S  2VLJE                  5    0.484  56.748   0.113   0.018   0.047   0.935
E P  2VLJE                  6    0.453  64.238  -0.803   0.058   0.017   0.925
E K  2VLJE                  7    0.472  96.988  -0.670   0.056   0.142   0.802
B Y  2VLJE                  8    0.337  71.910  -1.036   0.021   0.451   0.528
E L  2VLJE                  9    0.299  54.784   0.435   0.018   0.846   0.136
E F  2VLJE                 10    0.392  78.755  -0.697   0.018   0.846   0.136
E R  2VLJE                 11    0.294  67.372   0.550   0.021   0.756   0.223
E K  2VLJE                 12    0.442  90.981   0.869   0.004   0.514   0.481
E E  2VLJE                 13    0.538  94.006   0.785   0.004   0.085   0.910
E G  2VLJE                 14    0.697  54.885  -0.837   0.005   0.015   0.979
E Q  2VLJE                 15    0.449  80.245   0.612   0.004   0.197   0.799
E N  2VLJE                 16    0.550  80.447   1.343   0.003   0.718   0.279
B V  2VLJE                 17    0.070  10.820  -0.006   0.001   0.959   0.040
E T  2VLJE                 18    0.428  59.322   1.499   0.001   0.959   0.040
B L  2VLJE                 19    0.057  10.510   0.511   0.001   0.959   0.040
E S  2VLJE                 20    0.393  46.106   1.348   0.001   0.959   0.040
B C  2VLJE                 21    0.035   4.858  -0.367   0.001   0.959   0.040
E E  2VLJE                 22    0.442  77.200   1.484   0.001   0.959   0.040
B Q  2VLJE                 23    0.257  45.882  -0.666   0.001   0.900   0.099
B N  2VLJE                 24    0.271  39.689  -0.798   0.003   0.718   0.279
E L  2VLJE                 25    0.440  80.582  -1.368   0.004   0.138   0.858
E N  2VLJE                 26    0.595  87.079  -1.834   0.005   0.015   0.979
E H  2VLJE                 27    0.298  54.170  -0.561   0.005   0.045   0.951
B D  2VLJE                 28    0.279  40.276  -0.773   0.005   0.336   0.660
B A  2VLJE                 29    0.107  11.758   0.446   0.003   0.718   0.279
B M  2VLJE                 30    0.026   5.123   0.598   0.011   0.918   0.071
B Y  2VLJE                 31    0.071  15.173   0.578   0.011   0.918   0.071
B W  2VLJE                 32    0.032   7.720   0.482   0.011   0.918   0.071
B Y  2VLJE                 33    0.045   9.531   0.577   0.011   0.918   0.071
B R  2VLJE                 34    0.153  35.037   0.913   0.018   0.846   0.136
B Q  2VLJE                 35    0.113  20.218   0.455   0.022   0.552   0.426
E D  2VLJE                 36    0.423  60.969   1.575   0.004   0.085   0.910
E P  2VLJE                 37    0.491  69.645  -0.004   0.005   0.015   0.979
E G  2VLJE                 38    0.820  64.565  -1.826   0.003   0.003   0.994
E Q  2VLJE                 39    0.443  79.084   0.523   0.018   0.019   0.964
E G  2VLJE                 40    0.330  25.995  -0.510   0.018   0.088   0.893
B L  2VLJE                 41    0.088  16.149   0.569   0.005   0.336   0.660
B R  2VLJE                 42    0.231  52.945   0.326   0.002   0.816   0.182
B L  2VLJE                 43    0.082  15.087   0.412   0.001   0.959   0.040
B I  2VLJE                 44    0.020   3.681   1.310   0.001   0.959   0.040
B Y  2VLJE                 45    0.028   5.898   0.344   0.000   0.983   0.017
B Y  2VLJE                 46    0.062  13.292   0.408   0.000   0.983   0.017
B S  2VLJE                 47    0.067   7.888  -0.138   0.001   0.959   0.040
B Q  2VLJE                 48    0.206  36.738  -0.271   0.004   0.616   0.381
B I  2VLJE                 49    0.295  54.630  -1.435   0.020   0.205   0.775
E V  2VLJE                 50    0.549  84.427  -1.117   0.018   0.047   0.935
E N  2VLJE                 51    0.598  87.533  -1.249   0.018   0.047   0.935
E D  2VLJE                 52    0.424  61.070  -0.803   0.056   0.142   0.802
E F  2VLJE                 53    0.479  96.115  -0.810   0.064   0.216   0.721
E Q  2VLJE                 54    0.363  64.868  -0.162   0.019   0.141   0.840
E K  2VLJE                 55    0.595 122.474  -0.462   0.018   0.047   0.935
E G  2VLJE                 56    0.747  58.789  -1.817   0.018   0.019   0.964
E D  2VLJE                 57    0.581  83.679  -1.245   0.018   0.019   0.964
B I  2VLJE                 58    0.142  26.344   0.101   0.018   0.047   0.935
E A  2VLJE                 59    0.461  50.824   0.039   0.018   0.047   0.935
E E  2VLJE                 60    0.614 107.266  -1.055   0.004   0.085   0.910
B G  2VLJE                 61    0.235  18.487  -0.629   0.004   0.420   0.576
B Y  2VLJE                 62    0.066  14.168  -0.527   0.003   0.718   0.279
E S  2VLJE                 63    0.329  38.512   0.215   0.002   0.816   0.182
B V  2VLJE                 64    0.102  15.724  -1.848   0.001   0.900   0.099
E S  2VLJE                 65    0.354  41.465  -0.452   0.001   0.900   0.099
E R  2VLJE                 66    0.338  77.471  -0.007   0.003   0.718   0.279
E E  2VLJE                 67    0.523  91.438  -0.567   0.004   0.197   0.799
E K  2VLJE                 68    0.697 143.435  -1.010   0.005   0.015   0.979
E K  2VLJE                 69    0.705 144.977  -0.904   0.005   0.015   0.979
E E  2VLJE                 70    0.379  66.141  -0.438   0.004   0.197   0.799
E S  2VLJE                 71    0.358  41.946   0.996   0.003   0.718   0.279
B F  2VLJE                 72    0.046   9.312  -0.054   0.001   0.900   0.099
B P  2VLJE                 73    0.223  31.686   0.958   0.001   0.900   0.099
B L  2VLJE                 74    0.026   4.687   0.643   0.001   0.959   0.040
B T  2VLJE                 75    0.249  34.578   0.684   0.001   0.959   0.040
B V  2VLJE                 76    0.056   8.638   0.121   0.001   0.900   0.099
E T  2VLJE                 77    0.439  60.931   0.630   0.002   0.816   0.182
E S  2VLJE                 78    0.389  45.567  -0.804   0.004   0.616   0.381
B A  2VLJE                 79    0.167  18.392  -0.418   0.004   0.197   0.799
E Q  2VLJE                 80    0.474  84.674   0.804   0.004   0.138   0.858
E K  2VLJE                 81    0.488 100.279  -0.310   0.004   0.197   0.799
E N  2VLJE                 82    0.608  88.938  -0.713   0.018   0.047   0.935
B P  2VLJE                 83    0.242  34.283  -0.433   0.019   0.141   0.840
B T  2VLJE                 84    0.229  31.748   0.192   0.004   0.514   0.481
B A  2VLJE                 85    0.043   4.683  -1.137   0.001   0.900   0.099
B F  2VLJE                 86    0.114  22.980   1.162   0.001   0.959   0.040
B Y  2VLJE                 87    0.023   4.894   1.393   0.001   0.959   0.040
B L  2VLJE                 88    0.046   8.404   0.752   0.001   0.900   0.099
B C  2VLJE                 89    0.013   1.867   1.543   0.002   0.816   0.182
B A  2VLJE                 90    0.102  11.207   0.191   0.004   0.616   0.381
B S  2VLJE                 91    0.135  15.857  -0.205   0.004   0.420   0.576
B S  2VLJE                 92    0.261  30.636  -0.547   0.019   0.141   0.840
E S  2VLJE                 93    0.399  46.786  -1.493   0.018   0.047   0.935
E R  2VLJE                 94    0.406  92.997  -1.933   0.053   0.043   0.903
E S  2VLJE                 95    0.431  50.513  -1.607   0.053   0.043   0.903
E S  2VLJE                 96    0.442  51.838  -1.302   0.053   0.043   0.903
E Y  2VLJE                 97    0.357  76.376  -1.517   0.018   0.047   0.935
E E  2VLJE                 98    0.329  57.441  -1.406   0.018   0.047   0.935
E Q  2VLJE                 99    0.399  71.261  -1.252   0.018   0.088   0.893
E Y  2VLJE                100    0.385  82.317  -1.106   0.018   0.088   0.893
E F  2VLJE                101    0.368  73.817  -0.974   0.018   0.088   0.893
E G  2VLJE                102    0.341  26.837  -1.487   0.019   0.141   0.840
E P  2VLJE                103    0.330  46.813  -1.294   0.004   0.085   0.910
E G  2VLJE                104    0.456  35.911  -0.989   0.004   0.197   0.799
E T  2VLJE                105    0.334  46.354  -1.015   0.004   0.197   0.799
E R  2VLJE                106    0.342  78.295  -0.288   0.005   0.262   0.733
B L  2VLJE                107    0.190  34.844  -1.952   0.004   0.420   0.576
E T  2VLJE                108    0.314  43.524  -0.838   0.004   0.616   0.381
B V  2VLJE                109    0.111  17.091  -2.290   0.022   0.552   0.426
B T  2VLJE                110    0.222  30.861  -0.947   0.022   0.359   0.619
E E  2VLJE                111    0.388  67.819  -1.881   0.020   0.205   0.775
E D  2VLJE                112    0.550  79.241  -1.822   0.018   0.088   0.893
E L  2VLJE                113    0.421  77.122  -1.683   0.018   0.047   0.935
E K  2VLJE                114    0.407  83.658  -1.895   0.018   0.047   0.935
E N  2VLJE                115    0.535  78.353  -1.818   0.018   0.047   0.935
E V  2VLJE                116    0.424  65.200  -1.587   0.018   0.047   0.935
E F  2VLJE                117    0.362  72.694  -1.823   0.018   0.047   0.935
E P  2VLJE                118    0.372  52.773  -1.797   0.005   0.045   0.951
B P  2VLJE                119    0.336  47.636  -2.345   0.004   0.085   0.910
E E  2VLJE                120    0.328  57.319  -0.869   0.022   0.359   0.619
B V  2VLJE                121    0.205  31.585  -1.361   0.022   0.359   0.619
B A  2VLJE                122    0.260  28.597  -0.957   0.022   0.359   0.619
B V  2VLJE                123    0.212  32.569  -1.878   0.022   0.359   0.619
B F  2VLJE                124    0.194  38.835  -0.959   0.021   0.279   0.699
E E  2VLJE                125    0.323  56.446  -1.383   0.018   0.088   0.893
B P  2VLJE                126    0.122  17.326  -2.892   0.018   0.019   0.964
B S  2VLJE                127    0.246  28.819  -1.583   0.018   0.088   0.893
E E  2VLJE                128    0.409  71.452  -1.759   0.020   0.205   0.775
E A  2VLJE                129    0.450  49.634  -2.094   0.020   0.205   0.775
E E  2VLJE                130    0.526  91.927  -2.416   0.020   0.205   0.775
B I  2VLJE                131    0.272  50.264  -1.940   0.004   0.085   0.910
E S  2VLJE                132    0.529  61.952  -0.431   0.005   0.015   0.979
E H  2VLJE                133    0.557 101.391  -0.971   0.005   0.015   0.979
E T  2VLJE                134    0.722 100.114  -1.423   0.003   0.003   0.994
E Q  2VLJE                135    0.556  99.284  -0.988   0.005   0.045   0.951
E K  2VLJE                136    0.390  80.161   0.857   0.004   0.420   0.576
B A  2VLJE                137    0.065   7.196   0.192   0.001   0.900   0.099
B T  2VLJE                138    0.141  19.598   0.533   0.001   0.959   0.040
B L  2VLJE                139    0.032   5.932   0.298   0.001   0.959   0.040
B V  2VLJE                140    0.029   4.442   0.508   0.001   0.959   0.040
B C  2VLJE                141    0.014   1.909   1.464   0.001   0.959   0.040
B L  2VLJE                142    0.075  13.824   0.468   0.001   0.959   0.040
B A  2VLJE                143    0.026   2.854   0.024   0.001   0.900   0.099
B T  2VLJE                144    0.172  23.856   0.181   0.003   0.718   0.279
E G  2VLJE                145    0.336  26.451  -0.873   0.005   0.336   0.660
B F  2VLJE                146    0.323  64.866  -1.701   0.004   0.197   0.799
E Y  2VLJE                147    0.333  71.141  -1.134   0.004   0.138   0.858
E P  2VLJE                148    0.430  61.031  -2.111   0.004   0.085   0.910
E D  2VLJE                149    0.554  79.774  -2.476   0.004   0.085   0.910
E H  2VLJE                150    0.381  69.268  -1.214   0.005   0.336   0.660
B V  2VLJE                151    0.110  16.830  -0.306   0.003   0.718   0.279
B E  2VLJE                152    0.226  39.482   0.467   0.001   0.900   0.099
B L  2VLJE                153    0.042   7.672  -0.102   0.001   0.959   0.040
B S  2VLJE                154    0.175  20.522   0.250   0.001   0.959   0.040
B W  2VLJE                155    0.060  14.430   0.076   0.006   0.962   0.032
E W  2VLJE                156    0.272  65.368   0.692   0.001   0.959   0.040
E V  2VLJE                157    0.298  45.741   0.359   0.002   0.816   0.182
E N  2VLJE                158    0.641  93.784  -0.067   0.018   0.047   0.935
E G  2VLJE                159    0.718  56.483  -1.500   0.005   0.015   0.979
E K  2VLJE                160    0.625 128.583  -1.019   0.004   0.420   0.576
E E  2VLJE                161    0.425  74.213  -0.751   0.004   0.420   0.576
E V  2VLJE                162    0.345  52.980  -0.715   0.022   0.359   0.619
E H  2VLJE                163    0.442  80.345  -1.513   0.022   0.359   0.619
E S  2VLJE                164    0.547  64.132  -1.076   0.020   0.205   0.775
E G  2VLJE                165    0.342  26.884  -1.948   0.020   0.205   0.775
E V  2VLJE                166    0.318  48.923  -1.524   0.021   0.451   0.528
E S  2VLJE                167    0.432  50.677  -1.299   0.022   0.552   0.426
E T  2VLJE                168    0.439  60.917  -1.163   0.021   0.451   0.528
E D  2VLJE                169    0.330  47.510  -0.861   0.020   0.205   0.775
E P  2VLJE                170    0.423  60.038  -1.270   0.019   0.141   0.840
E Q  2VLJE                171    0.504  90.086  -0.941   0.020   0.205   0.775
E P  2VLJE                172    0.379  53.766  -0.867   0.021   0.451   0.528
B L  2VLJE                173    0.207  37.828  -1.104   0.022   0.552   0.426
E K  2VLJE                174    0.493 101.451  -1.005   0.021   0.451   0.528
E E  2VLJE                175    0.403  70.422  -1.947   0.020   0.205   0.775
E Q  2VLJE                176    0.553  98.837  -1.261   0.019   0.141   0.840
E P  2VLJE                177    0.487  69.063  -2.693   0.021   0.279   0.699
E A  2VLJE                178    0.474  52.257  -1.850   0.022   0.359   0.619
E L  2VLJE                179    0.413  75.620  -1.685   0.022   0.359   0.619
E N  2VLJE                180    0.515  75.425  -1.844   0.020   0.205   0.775
E D  2VLJE                181    0.652  93.910  -2.717   0.005   0.045   0.951
E S  2VLJE                182    0.398  46.587  -1.875   0.004   0.085   0.910
E R  2VLJE                183    0.306  70.005   0.248   0.004   0.514   0.481
B Y  2VLJE                184    0.118  25.131  -1.132   0.002   0.816   0.182
B S  2VLJE                185    0.099  11.556  -1.251   0.001   0.900   0.099
B L  2VLJE                186    0.101  18.548  -1.107   0.001   0.900   0.099
B S  2VLJE                187    0.088  10.267  -0.660   0.001   0.959   0.040
B S  2VLJE                188    0.043   4.993  -1.309   0.001   0.900   0.099
B R  2VLJE                189    0.160  36.548  -0.022   0.001   0.959   0.040
B L  2VLJE                190    0.042   7.727  -0.105   0.001   0.900   0.099
E R  2VLJE                191    0.265  60.777   0.796   0.001   0.900   0.099
B V  2VLJE                192    0.048   7.362   0.355   0.002   0.816   0.182
E S  2VLJE                193    0.326  38.195   1.038   0.021   0.451   0.528
E A  2VLJE                194    0.304  33.468  -0.709   0.020   0.205   0.775
E T  2VLJE                195    0.567  78.671  -1.038   0.064   0.216   0.721
E F  2VLJE                196    0.428  85.859  -0.104   0.020   0.205   0.775
B W  2VLJE                197    0.163  39.105  -1.806   0.020   0.205   0.775
E Q  2VLJE                198    0.597 106.696  -0.356   0.020   0.205   0.775
E N  2VLJE                199    0.450  65.807   0.263   0.005   0.045   0.951
E P  2VLJE                200    0.523  74.185  -1.283   0.005   0.015   0.979
E R  2VLJE                201    0.702 160.735   0.297   0.005   0.015   0.979
E N  2VLJE                202    0.377  55.164   1.092   0.005   0.262   0.733
E H  2VLJE                203    0.359  65.357   1.676   0.002   0.816   0.182
B F  2VLJE                204    0.060  12.062   1.073   0.001   0.959   0.040
E R  2VLJE                205    0.298  68.128   1.944   0.001   0.959   0.040
B C  2VLJE                206    0.022   3.145   0.902   0.001   0.959   0.040
B Q  2VLJE                207    0.252  45.061   1.143   0.001   0.959   0.040
B V  2VLJE                208    0.076  11.743  -0.182   0.001   0.900   0.099
E Q  2VLJE                209    0.482  86.121   1.130   0.001   0.959   0.040
B F  2VLJE                210    0.167  33.577   0.532   0.002   0.816   0.182
E Y  2VLJE                211    0.614 131.319  -0.026   0.004   0.138   0.858
E G  2VLJE                212    0.769  60.552  -1.230   0.005   0.045   0.951
E L  2VLJE                213    0.552 101.035  -0.877   0.004   0.138   0.858
E S  2VLJE                214    0.468  54.850  -1.061   0.004   0.197   0.799
E E  2VLJE                215    0.560  97.849  -0.046   0.004   0.420   0.576
E N  2VLJE                216    0.553  80.886   0.233   0.004   0.616   0.381
B D  2VLJE                217    0.200  28.834   0.070   0.003   0.718   0.279
E E  2VLJE                218    0.638 111.441   0.114   0.002   0.816   0.182
B W  2VLJE                219    0.219  52.621   0.926   0.002   0.816   0.182
E T  2VLJE                220    0.532  73.747  -0.282   0.018   0.846   0.136
E Q  2VLJE                221    0.277  49.454   0.218   0.022   0.552   0.426
E D  2VLJE                222    0.612  88.189  -0.862   0.021   0.279   0.699
E R  2VLJE                223    0.498 114.042  -1.044   0.018   0.088   0.893
E A  2VLJE                224    0.479  52.786  -1.352   0.018   0.088   0.893
E K  2VLJE                225    0.632 129.900   0.144   0.005   0.045   0.951
E P  2VLJE                226    0.322  45.635  -1.211   0.004   0.085   0.910
E V  2VLJE                227    0.544  83.628  -0.182   0.004   0.197   0.799
E T  2VLJE                228    0.341  47.269  -0.298   0.004   0.420   0.576
E Q  2VLJE                229    0.386  68.993   0.100   0.004   0.514   0.481
E I  2VLJE                230    0.402  74.444  -0.736   0.003   0.718   0.279
B V  2VLJE                231    0.197  30.233   0.688   0.003   0.718   0.279
E S  2VLJE                232    0.289  33.824  -0.304   0.021   0.756   0.223
E A  2VLJE                233    0.323  35.584  -0.087   0.021   0.756   0.223
E E  2VLJE                234    0.379  66.229   0.028   0.018   0.846   0.136
B A  2VLJE                235    0.246  27.109  -0.431   0.018   0.846   0.136
B W  2VLJE                236    0.269  64.574   0.109   0.021   0.756   0.223
E G  2VLJE                237    0.362  28.466  -2.325   0.004   0.616   0.381
E R  2VLJE                238    0.423  96.890  -0.489   0.022   0.359   0.619
E A  2VLJE                239    0.409  45.039  -2.531   0.019   0.141   0.840
E D  2VLJE                240    0.878 126.462  -0.334   0.003   0.003   0.994

Explain the output.  Go back.
```

---
